# A Safe Place to Learn: Peer Research Qualitative Investigation of gameChange Virtual Reality Therapy

**DOI:** 10.2196/38065

**Published:** 2023-01-16

**Authors:** Jessica Bond, Alexandra Kenny, Vanessa Pinfold, Lisa Couperthwaite, The gameChange Lived Experience Advisory Panel, Thomas Kabir, Michael Larkin, Ariane Beckley, Laina Rosebrock, Sinéad Lambe, Daniel Freeman, Felicity Waite, Dan Robotham

**Affiliations:** 1 McPin Foundation London United Kingdom; 2 School of Psychology Aston University Birmingham United Kingdom; 3 Department of Psychiatry University of Oxford Oxford United Kingdom; 4 Oxford Health National Health Service Foundation Trust Oxford United Kingdom; 5 National Institute for Health and Care Research Oxford Health Biomedical Research Centre Oxford United Kingdom

**Keywords:** peer research, lived experience, qualitative methods, interpretative phenomenological analysis, IPA, anxiety, psychosis, virtual reality, VR, cognitive therapy, automated, implementation

## Abstract

**Background:**

Automated virtual reality (VR) therapy has the potential to substantially increase access to evidence-based psychological treatments. The results of a multicenter randomized controlled trial showed that gameChange VR cognitive therapy reduces the agoraphobic avoidance of people diagnosed with psychosis, especially for those with severe avoidance.

**Objective:**

We set out to use a peer research approach to explore participants’ experiences with gameChange VR therapy. This in-depth experiential exploration of user experience may inform the implementation in clinical services and future VR therapy development.

**Methods:**

Peer-led semistructured remote interviews were conducted with 20 people with a diagnosis of psychosis who had received gameChange as part of the clinical trial (ISRCTN17308399). Data were analyzed using interpretative phenomenological analysis and template analyses. A multiperspectival approach was taken to explore subgroups. Credibility checks were conducted with the study Lived Experience Advisory Panel.

**Results:**

Participants reported the substantial impact of anxious avoidance on their lives before the VR intervention, leaving some of them housebound and isolated. Those who were struggling the most with agoraphobic avoidance expressed the most appreciation for, and gains from, the gameChange therapy. The VR scenarios provided “a place to practise.” Immersion within the VR scenarios triggered anxiety, yet participants were able to observe this and respond in different ways than usual. The “security of knowing the VR scenarios are not real” created a safe place to learn about fears. The “balance of safety and anxiety” could be calibrated to the individual. The new learning made in VR was “taken into the real world” through practice and distilling key messages with support from the delivery staff member.

**Conclusions:**

Automated VR can provide a therapeutic simulation that allows people diagnosed with psychosis to learn and embed new ways of responding to the situations that challenge them. An important process in anxiety reduction is enabling the presentation of stimuli that induce the original anxious fears yet allow for learning of safety. In gameChange, the interaction of anxiety and safety could be calibrated to provide a safe place to learn about fears and build confidence. This navigation of therapeutic learning can be successfully managed by patients themselves in an automated therapy, with staff support, that provides users with personalized control. The clinical improvements for people with severe anxious avoidance, the positive experience of VR, and the maintenance of a sense of control are likely to facilitate implementation.

## Introduction

### Background

gameChange is an automated virtual reality (VR) cognitive therapy designed to reduce agoraphobic avoidance and distress in everyday situations. In a multicenter randomized controlled trial with 346 people with psychosis, we found that gameChange led to significant reductions in avoidance and distress [1]. Overall, there were small effect size improvements in agoraphobic avoidance, agoraphobic distress, and perceptions of recovery. However, patients with the most severe agoraphobia, for example, those struggling to leave the home, had large effect size improvements in agoraphobic distress and avoidance as well as significant improvements in persecutory ideation, quality of life, and perceived recovery, which were maintained at 6 months.

Agoraphobic avoidance is understood from a cognitive perspective in gameChange. The avoidance is a defense (or safety-seeking) behavior in response to fearful thoughts [2,3]. Fearful thoughts often develop because of past unpleasant or threatening experiences. However, the fears are maintained as the absence of harm in the present moment is attributed to the use of defenses rather than there being no current threat. Even when in the anxiety-provoking situations, the use of more subtle defenses (eg, rushing or avoiding eye contact) plays the same maintaining role for catastrophic cognitions [4]. In treatment, defenses must be dropped so that the threat beliefs can be evaluated fully. This is not to overlook the fact that many patients with psychosis have had difficult experiences and may currently be living in adverse circumstances and potentially facing ongoing threats. Although there may not be current overt actions against the person, there can be more implicit and subtle forms of threat, particularly linked to stigma. Therefore, clinicians working with patients should always be aware of the potential for actual threat and assess appropriately.

On the basis of cognitive psychology principles, gameChange VR therapy was developed through a user-centered design process involving >500 hours of input from people with lived experience [5,6]. In 6 sessions, people enter interactive computer-generated simulations of everyday scenarios such as a café, a bus, or a physician’s waiting room. Guided by a virtual coach, patients are encouraged to experiment with dropping defense behaviors and to evaluate their fears. A member of the staff is present to assist with the equipment and help plan homework tasks to consolidate the learning from VR. Consistent with the cognitive model, we found in the trial that improvements in agoraphobic anxiety and avoidance were mediated by reductions in both a wide range of threat cognitions and the use of within-situation defense behaviors [1].

### Objectives

In this study, we used a peer research approach to try to understand the participant experience of gameChange VR therapy in depth. Peer research is steered and conducted by people with relevant lived experiences. Peer methods have the potential to facilitate greater depth and more nuanced data collection and analysis by enhancing rapport [7] and leveling power [8]. Working collaboratively with researchers from different backgrounds, including lived experience, can enhance the validity and trustworthiness of qualitative analysis [9,10]. Interpretative phenomenological analysis (IPA) is a qualitative approach that focuses on participants’ lived experiences and how they make sense of them [11,12]. IPA was chosen as it explicitly acknowledges the role of the researcher in the interpretation, which complements the peer research approach of valuing the insights that lived experience can bring [11]. Template analysis can be used to extend and develop IPAs for larger samples [13]. We conducted a peer-methods qualitative study incorporating IPA and template analysis to explore participants’ experiences with gameChange.

## Methods

### Overview

This was a qualitative peer-methods study investigating the experiences with gameChange VR therapy for agoraphobic avoidance. A multiperspectival design was used [14], and a peer research approach was taken, including collaboration with the gameChange Lived Experience Advisory Panel (LEAP). The LEAP was a panel of 12 people with lived experience of psychosis. Full details of the study methodology are provided in the published study protocol [15]. Criteria to ensure credibility in qualitative research [16,17] were used to inform the design and ensure rigor in the conduct of the study.

### Ethics Approval

The study received Health Research Authority approval and Health and Care Research Wales approval (IRAS 256895), as well as ethics approval from the National Health Service (NHS) South Central - Oxford B Research Ethics Committee (19/SC/0075) as part of the gameChange trial.

### Research Team

People with lived experience of psychosis and anxious social avoidance were involved in all stages of the study. The research team included peer researchers who had had similar experiences to those being explored, a qualitative researcher with lived experience of mental health issues that did not directly overlap with those of the participants, clinical psychologists who designed and used VR therapy, and a qualitative researcher who pioneered the IPA approach. The LEAP, facilitated by the McPin Foundation, a charity that promotes the use of lived experience in research, contributed to the design, conduct, and analysis of the study. We aimed to use the peer knowledge within the study at all stages, including the design, data collection, analysis, and presentation of the findings.

### Participants

We recruited participants who had received gameChange VR therapy as part of the trial (ISRCTN17308399). The participants were people accessing NHS mental health services with a diagnosis of psychosis and self-reported difficulties going into everyday social situations because of anxiety. The inclusion and exclusion criteria for the trial are listed in the trial protocol [18]. The additional inclusion criteria for this qualitative study were willingness and ability to provide informed consent to participate in the interview and willingness to have the interview audio recorded.

### Sampling and Recruitment

Consistent with IPA principles, the recruitment procedure sought homogeneity—the locus of homogeneity in this study was receipt of gameChange therapy. The primary sampling method was to invite a consecutive cohort of people in the final phase of the trial between April 2021 and September 2021. To explore different experiences of treatment using a multiperspectival design, we sought a larger sample size (N=20), including participants with low, medium, and high uptake of therapy. When possible, this was supplemented with purposive sampling to ensure variation across demographic characteristics (including age, gender, and ethnicity) and trial centers. The recruitment strategy was revised because of the high uptake of therapy in the trial (just under 90% of participants received a dose of therapy, which was defined as a minimum of 3 sessions). Therefore, the subgroups for the multiperspectival design were derived retrospectively from the data and are presented in the *Results* section.

A total of 27 people were invited to participate, of whom 7 (26%) declined and 20 (74%) consented. Participants were from each of the 5 trial centers that recruited participants from NHS mental health trusts across England: Avon and Wiltshire Mental Health Partnership NHS Trust; Greater Manchester Mental Health NHS Foundation Trust; Cumbria, Northumberland, Tyne, and Wear NHS Foundation Trust; Nottinghamshire Healthcare NHS Foundation Trust; and Oxford Health NHS Foundation Trust. Recruitment was facilitated by the local trial coordinator. Written or audio informed consent, including consent to the use of pseudonymized quotes, was obtained from all participants.

### Procedure

The semistructured interview guide (Multimedia Appendix 1) was developed in collaboration with the LEAP, with reference to existing literature, and in line with the principles of IPA [19-21]. The guide was refined following pilot interviews with members of the LEAP. The guide was used flexibly, with a focus on eliciting the participants’ own account. This involved using open-ended descriptive and narrative questions to explore the context, followed by individually tailored prompts to elicit further information or clarification and using the participants’ own language whenever possible. The focus of the interview guide was on participants’ experience with the gameChange therapy. It included questions such as *What is your experience of going out of the house or going into social situations?* *What was it like to be in everyday situations in virtual reality?* and *Compared to what you told us about your life before gameChange, is life different in any way now? How so?*

Interviews were conducted between April 2021 and September 2021. The first 40% (8/20) of interviews were conducted jointly by JB and AK, a further 55% (11/20) were facilitated by AK alone, and 5% (1/20) were facilitated by JB alone. Interviews were conducted remotely, either by phone (14/20, 70%) or web-based video call (6/20, 30%). The mean duration was 80 (SD 22; range 40-105) minutes, with most (13/20, 65%) completed over 2 sessions. The peer researcher chose when, how, and what to disclose about their related mental health experiences, and this differed between interviews. Both interviewers kept field notes, including on the use and impact of sharing the peer identity. Interviews were audio recorded, transcribed verbatim, and deidentified.

### Analysis

A total of 25% (5/20) of the transcripts were coded using IPA [11,20]. IPA focuses on participants’ lived experiences and how they make sense of them [11]. The remaining transcripts were analyzed using the initial IPA themes as a preliminary *template*. This work was conducted using template analysis [22], which can be used with IPA to analyze larger samples [13].

The multiperspectival design provides a structure for exploring both the individual account and related groups [14]. In our study, the subsamples were identified via preliminary analysis rather than via sampling strategies. Four subsamples related to overall experience and the impact of therapy were identified: (1) *Big changes* (4/20, 20%), (2) *Better ways to cope* (9/20, 45%), (3) *Hard to say or hard to hang on to* (5/20, 25%), and (4) *Struggling on* (2/20, 10%).

The 25% (5/20) of transcripts included in the IPA analysis were selected based on the richness of the data and to ensure a range of trial sites and ages and a balance of gender. To maintain the homogeneity of the sample, a requirement of IPA, the 5 transcripts expressed a similar perspective on gameChange. The analytical procedure for IPA followed the approach outlined by Smith et al [11,20]. This included line-by-line annotation of each transcript, mapping what mattered to each participant (“objects of concern”) with the meaning assigned to the experience (“experiential statements”). Meanings were clustered and interpreted for each case. Across cases, themes were identified that encompassed the phenomenological experiences and understanding of the 25% (5/20) of participants, forming superordinate and subordinate themes. IPA provided the foundation for the subsequent template analysis.

A provisional *template* was developed from the IPA themes and used to initiate the template analysis of the remaining data. The template analysis followed the steps outlined by King et al [22]. In line with the multiperspectival design [14], the transcripts were introduced in subgroups, focusing on each in turn. Analyzing the data in these batches made comparisons between perspectives starker. New codes were assigned and the template was updated at least once after each group was added. Once all data had been analyzed, the template was reviewed against all transcripts. Two analysis sessions were held with the LEAP to develop the template. LEAP members were given an overview of the themes and shown a selection of quotes. The focus was on identifying patterns within the data, resolving ambiguity in the accounts, and considering how the participants’ experiences resonated with their own.

The IPA analysis was conducted by JB, AK, and LC. DR and VP contributed to the template analysis. Supervisory support was provided at each stage of the analysis by ML and FW.

### Reflexivity and Credibility

All members of the research team reflected on the different perspectives that they brought to the study design, setup, data collection, and analysis. For example, in the analysis, the peer researchers drew on their lived experience to support their interpretation of meaning in the data; this was particularly relevant when exploring meanings related to participants’ experiences of anxious avoidance and psychosis.

Credibility checks were conducted with members of the gameChange LEAP. Their input was used to corroborate and deepen the interpretation of the themes. The reflexive logs, transcripts, interview schedule development, and minutes of supervisory research team meetings provided an audit trail for analysis. Illustrative quotes, description of the study context, and participant characteristics allowed for an assessment of the transferability of the findings to other settings and contexts.

## Results

### Contextualizing the Data

A total of 20 people participated: 11 (55%) women and 9 (45%) men. The average age of the participants was 37 (SD 12.9) years. In total, 85% (17/20) of the participants identified as White British individuals, 10% (2/20) identified as British Asian individuals, and 5% (1/20) identified as Polish. As can be seen in Table 1, most (15/20, 75%) participants were unemployed. A total of 20% (4/20) of the participants said that they spent a lot of time playing computer games and described themselves as “gamers.” In total, 60% (12/20) of the participants attended 6 sessions of therapy, and 40% (8/20) attended 7 sessions. For 55% (11/20) of the participants, these VR sessions were facilitated by a clinical psychologist; for 30% (6/20) of the participants, it was an assistant psychologist; and 15% (3/20) of the participants worked with peer support workers. The participant characteristics in this qualitative study were consistent with those in the main trial (see the trial outcome report [1]).

**Table 1:** Participant characteristics (N=20).

Demographic and clinical characteristics		Participants, n (%)
**Marital status**		
	Single	15 (75)
	Married	3 (15)
	Divorced	1 (5)
	Cohabiting	1 (5)
**Employment**		
	Unemployed	15 (75)
	Retired	2 (10)
	Carer	1 (5)
	Employed (full-time)	1 (5)
	Student (part-time)	1 (5)
**Living situation**		
	Living with parents or another relative	8 (40)
	Living alone	7 (35)
	Living with others	4 (20)
	Living with a spouse or partner	1 (5)
**Mental health service context at the time of interview**		
	CMHT^a^	15 (75)
	EIP^b^ team	5 (25)

^a^CMHT: community mental health team.

^b^EIP: early intervention in psychosis.

### Overview of Findings

An overview of the key findings is presented. This is followed by a description of the subgroups identified for the multiperspectival design. The cross-case analysis of participants’ experiences with the intervention is then presented. Illustrative quotes, with details omitted to protect anonymity, are presented in the text and in Multimedia Appendix 2.

#### Summary of Key Findings

There were 5 superordinate themes (Textbox 1). First, anxious avoidance carries a cost. Participants described living restricted lives in which fears made it hard to do everyday activities. Second, participants shared their curiosity and motivation to try VR therapy. Third, VR therapy provided a place to learn about anxiety and practice different or new responses. Fourth, the VR simulations triggered an anxiety response, yet participants simultaneously recognized that the environments were largely safe. This interaction of anxiety and safety could be calibrated by the individual to provide a safe place to learn about fears and build confidence. Finally, VR was a training ground to do things differently. Through practice and conversations with the member of the staff who was present, participants took the key learning from VR into the real-world situations that mattered to them.

**Overview of superordinate and subordinate themes.**

- Experience and cost of anxious avoidance
- Reasons to try: curiosity and motivation
- A place to practice
  An immersive experience
  A chance to observe anxiety
  New ways of responding
- The security of knowing it is not real
  The sweet spot of safety and anxiety
  Calibrating for a personalized approach
- Taking it into the real world
  From training wheels to real-world practice
  One thing to hold onto

### The 4 Perspectives

#### Overview of the 4 Perspectives

We identified 4 subgroups of participants based on the descriptions of their experience and impact of the gameChange therapy. The descriptions varied from life-changing to experiencing little change, although all described gameChange as a valuable experience. Illustrative quotes for each subgroup are presented in Table 2.

**Table 2:** Illustrative quotes from the participant subgroups (N=20).

Subgroup	Participants, n (%)	Illustrative quotes
Big changes	4 (20)	“It’s very different now, I can open my curtains and look out my window, I can go out to local shops and places, I can meet up with friends and family, I can do such a lot more.” [Participant 27] “Before I would never do that, ever. But I did that and I was talking to new people and I was having a great time. It’s just being in social situations instead of hiding from them, I’m just trying to embrace them.” [Participant 8]
Better ways to cope	9 (45)	“It definitely helped with anxiety. I still suffer from some of the things I did, well, all of the things I did before, but it’s definitely, definitely helped it in some way.” [Participant 3] “Before I’m just looking at the floor wherever I’m going but now I’m looking at everyone, like, in their eyes and stuff. Even though I’m still too scared to go to the supermarket on my own, I have been able to walk past it on my own, like walk up there and past it, whereas before I wouldn’t have been able to do that. I’ve been in small shops on my own and the bus, I can do that on my own now without worrying.” [Participant 25]
Hard to say or hard to hang onto	5 (25)	“Now because my mood is quite good, I’m thinking, ‘Yeah, actually. I did take away stuff.’ But when I’m really anxious, like I have been really anxious over the last couple of weeks...and then I’m like, I haven’t learnt anything.” [Participant 17] “I mean I wasn’t too bad with a lot of the situations anyway, so I don’t know if I’ve taken a lot from it, but I’d like to think I have but I’m not entirely sure. I’m not entirely sure if it’s had any massively notable impact on me.” [Participant 13]
Struggling on	2 (10)	“It has not really made a magical difference for me, unfortunately, but I can see how it can be very helpful. I think I’m just too entrenched in my avoidance little bubble, that it wasn’t strong enough to pull me out of it. It’s just the same.” [Participant 19] “Like, I say, still go to cafes, still get on the bus, go shopping. I didn’t go food shopping yesterday. I was really, really anxious yesterday in town with the boys. I couldn’t do my food shopping. My body wouldn’t let me do it. My mind affects my body.” [Participant 26]

#### Big Changes

A total of 20% (4/20) of the participants described gameChange as a transformative experience. Participants in this group marveled at how far they had come—they were able to do things they had not imagined at the start of therapy, when they were often housebound and experiencing very low mood. These accounts were characterized by excitement and enthusiasm.

#### Better Ways to Cope

A total of 45% (9/20) of the participants described experimenting with new ways to approach and cope with situations despite some ongoing anxiety. For these participants, social situations were less stressful but not yet comfortable.

#### Hard to Say or Hard to Hang Onto

A total of 25% (5/20) of the participants noted some small changes following therapy but found these hard to identify when asked directly or hard to maintain when anxiety increased or mood dipped. Participants valued aspects of the therapy but did not necessarily find that the VR scenarios triggered their anxiety. Participants in this group were often able to engage in some social situations before starting therapy.

#### Struggling On

In total, 10% (2/20) of the participants recognized the potential of the therapy but described little personal benefit as they struggled with psychotic experiences. They continued to feel anxious in the familiar and often essential (eg, shopping) situations they were able to engage in even before the start of therapy.

### Main Themes

#### The Experience and Cost of Anxious Avoidance

All participants spoke about the cost of anxious avoidance. Some participants described themselves as almost housebound, others avoided specific situations, and a few could perform essential tasks in an area where they felt safe. However, all felt the burden and restriction of anxious avoidance:

It affects my life big time because I can’t do half the things that I want to do or what a normal everyday person would do. I want to do something but sometimes it can hold me back and it can really affect the way you think, the way you act and the person that you are.

Participant 27, Big changes subgroup

As in the previous extract, participants shared the reasons for withdrawal: many felt threatened by others, at risk of physical or social harm, or that others were observing or talking about them. Some were anxious about showing symptoms in public as they feared that this would draw attention and invite judgment. Participants described the tactics that they had developed to try to reduce these risks, but some strategies had life-limiting effects. Some described their situation as intolerable, affecting self-esteem and quality of life, disrupting careers, impeding friendships, and making them feel “almost not like a human being.” Although all participants described life being “limited” by anxious avoidance, not all were distressed by inhabiting a small world.

#### Reasons to Try: Curiosity and Motivation

As VR therapy was new to the participants, most had no preconceived notions of what gameChange would be like or how it would help. Many said that they were “open-minded” to try gameChange. However, the reasons to engage differed:

I went in with an open mind. I was willing to give anything a go because I just didn’t want to be in the same state I was in after episode one.

Participant 3, Better ways to cope subgroup

I’m interested in technology, new technology. It’s quite exciting. So, that really encouraged me to get involved as well.

Participant 1, Big changes subgroup

As mentioned previously, for some, the opportunity appeared when they were ready to receive and engage with it. Others noted that experiencing a difficult time before gameChange fueled their desire for change. This was often accompanied by a sense of personal responsibility for the intervention’s success. Some were open to gameChange as they had been unwell for a long time and were keen to try anything that could alleviate their symptoms. Others simply saw it as an opportunity to do something in an otherwise restricted life: “I don’t really have anything to do so I was like: ‘Go on then, I might as well do it’” (participant 7, *Hard to say* subgroup).

The use of VR in gameChange was appealing to more than half (11/20, 55%) of the participants. Those who had heard of VR were attracted by its novelty, they were “interested” or “intrigued” to see what it would be like. They understood its therapeutic use as “a modern new way to treat social anxiety”:

I was, like, curious, I suppose, yeah. Its cutting-edge technology, isn’t it?

Participant 26, Struggling on subgroup

Of the 4 participants who identified as gamers, 3 (75%) were primarily attracted to the intervention as it was a chance to use VR.

#### A Place to Practice

I really enjoyed it. Well, I say enjoyed it, it was actually quite hard work. I found it very realistic when I went in, more than I expected, I think. And it was a lot harder than I thought it was going to be.

Participant 1, Big changes subgroup

Most (14/20, 70%) participants, including one of the 4 gamer participants, were surprised by how realistic it was. Participants seemed to be struck by its immersive quality:

Going into VR was like going into another world, kind of thing. It was really like everything was not realistic, you could tell it was all fake, but at the same time you felt like you were actually in there.

Participant 25, Better ways to cope subgroup

It was like being in a film, it was. You put the helmet [VR headset] on and you’re taken to another world.

Participant 26, Struggling on subgroup

Immersion was created by the graphics, background noise, and interactive details:

You can open the door. You can lift a can of beans up and put your bank card on the meter to pay for it. I thought it was amazing.

Participant 21, Better ways to cope subgroup

This immersion and perceived realism meant that many participants saw the VR scenarios as a meaningful environment to practice in. Participants who identified as gamers tended to be less impressed with the VR world.

Participants described developing new insights into their experience of anxiety, as described in the following extract:

I didn’t realise things like I was looking for an escape route and going to it. And now, I’m aware of that, I cannot do it so much. You know, it’s just sort of being aware.

Participant 1, Big changes subgroup

Being in the VR simulations could trigger anxiety. Participants spoke of sweaty palms and racing hearts and of realizing that they were unconsciously trying to use coping mechanisms such as looking for an escape route or exit, avoiding eye contact, or diminishing their physical presence. For some, this was a hard and shocking realization:

...that was really an eye-opener, and I was quite shocked at how it has affected my life for so long.

Participant 27, Big changes subgroup

VR provided an opportunity to observe anxiety and automatic responses with some emotional distance. It was “a buffer” or a chance “to step back and think about how they are reacting,” as members of the LEAP described it. Responses that were overwhelming and panicked in real life could be slowed, picked apart, and rerun. A LEAP member commented that VR was like a magnifying glass, clarifying the details and nuances of the situation. This led to participants developing greater understanding of what happens in moments of anxiety. This could be empowering and raised the potential to change these automatic responses.

Participants described VR as a place to practice new ways of responding in situations:

Make eye contact. I smile a bit more at people. Little things you don’t realise you haven’t been doing till you go and do the headset. It made me realise a lot, that I had just focused my life around the voices and not taken any notice of the outside world. That’s why I’ve been taking more notice of things when I’m out now.

Participant 23, Better ways to cope subgroup

The automated virtual coach and staff member made suggestions to drop defenses and try new strategies to build confidence. Despite the anxiety, it was safe to try new approaches as participants knew that the VR was not real.

#### “The Security of Knowing It Is Not Real”

The nature of VR means that, despite knowing that it is a simulation, reactions are typically real—as evidenced in the anxiety responses that the participants experienced:

Even though you’d tell yourself that you’re in a safe environment and the people weren’t going to necessarily react as you would expect a real-life person to react, your brain is still telling you that you’re in danger and you’re at risk here so yes, it did make you feel quite anxious.

Participant 2, Big changes subgroup

However, knowing that the VR simulations were not real allowed participants to feel a sense of safety:

It makes you face your fears but in a less extreme way because these people, you know aren’t real, but you still have the feeling of it being people.

Participant 16, Better ways to cope subgroup

Participants described an active interaction between safety and anxiety. For example, a sense of safety was required for participants to “slowly push themselves out of their comfort zone” (participant 8, *Big changes* subgroup), try the new ways of responding that the automated virtual coach and staff member suggested, and learn to tolerate discomfort. This was a delicate balance. If anxiety was “dialled” too low, VR was perceived as boring and ineffective. If safety was “dialled” too low (or anxiety too high), VR was intense and draining:

It’s a fine line: you want it to be realistic enough, so it’s a good practice, but it’s also quite helpful if it’s not totally realistic because then you get that security of knowing that it’s not real.

Participant 1, Big changes subgroup

As mentioned previously, participants understood the importance of locating this “sweet spot,” noting that it was sometimes helpful that the VR was not photorealistic or that they knew they were in no danger despite feeling bodily symptoms of anxiety. Finding the right balance could be difficult. For some participants, aspects of the VR scenarios matched real-world triggers, leading to fears or memories of past difficulties and resulting in spikes in anxiety. At these moments, it was sometimes hard to feel safe enough to create new learning in VR.

Levels of anxiety and safety could be calibrated by and to the individual through choice of the levels within the program or by the actions of the staff member:

The deliverer knew if I was putting defences up and she would tell me different ways to cope. There was one scenario where I was in the street and people walked past and I put my defences up because I kept looking at them walking past and my therapist said to relax and look at the cars and describe what’s in the cars and stuff.

Participant 11, Hard to say or hard to hang onto subgroup

The features of the software that often increased anxiety were “realness” and immersion in the scenarios, background noise, the scenarios becoming more crowded, and specific tasks such as waiting in a queue. Features that increased the sense of safety were that the characters and scenarios did not look as real as the real-world situations, the gradual increase in intensity of the levels, and having the option of removing the headset.

There were layers of support from the virtual coach, Nic, and the staff member. Those with higher baseline levels of anxiety described the coach as comforting, and participants appreciated her guidance and encouragement:

When I did a level, she would be like, “Well done, you did great.” She was good in that sense. It was someone familiar that I would see every time I went in.

Participant 25, Better ways to cope subgroup

Those who were more confident in VR described the coach as “repetitive,” “slow,” and at times “irritating.”

The staff member was able to build on the generic support that was programmed in the software through the virtual coach, increasing the participants’ sense of safety to facilitate more exploration and experimentation in VR. The staff member was attuned to the participants’ responses, suggesting pauses, encouraging repetition of scenarios or levels that they found challenging, or helping identify and drop specific defenses:

I think the deliverer was quite helpful in saying: “In that one you appeared more anxious, do you want to do that one again? You spoke a bit quietly in that one, did you want to do it again, you didn’t seem that confident doing that one, would you like to try it again?”

Participant 15, Better ways to cope subgroup

I would use coping strategies. I didn’t realise until the deliverer would tell me what I did—I didn’t recognise it myself—so, the deliverer would tell me certain things that would give me more confidence for the VR, but also, when leaving the session as well.

Participant 3, Better ways to cope subgroup

In this way, the sweet spot of anxiety and safety could usually be found for each person, creating the conditions for them to try new ways of responding in the VR scenario.

#### Taking It Into the Real World

The fear was there but what I was fearing didn’t actually happen which was good. That, for me, was part of the therapy in the sense that it made you realise that what you fear is going to happen isn’t necessarily what will happen. It made you re-evaluate if you like your way of thinking.

Participant 2, Big changes subgroup

Participants understood that VR was a safe place to do things that scared them—“training wheels” where they could take “baby steps”—before translating them to the real world:

The everyday situations that I found difficult, the more I practised them in the VR, the more I could get confident and be more confident in day-to-day life. I think because I was learning about it in the VR and practising and practising and practising, I could then take that and build up more confidence and do it in the everyday real world.

Participant 27, Big changes subgroup

I guess it’s like the training wheels, isn’t it? You get used to a situation simulating it and then go and try it in the real world.

Participant 5, Hard to say or hard to hang onto subgroup

Many participants recognized the need to practice between VR sessions and appreciated being given “homework.” Some acknowledged that this structure helped them push themselves. Others found additional ways to reinforce learning, such as writing notes and asking questions, periods of active reflection, and debriefing with the deliverer and other people. Some used sessions with their care teams to implement homework tasks. These participants were not deterred by circumstances such as COVID-19 restrictions hindering what they could do in the real world, and they were able to adjust the tasks around them. These participants tended to describe gaining the most benefit from the therapy and were in the *Big changes* and *Better ways to cope* subgroups.

Participants needed motivation to face anxiety-provoking situations—the potential rewards needed to be high enough for the effortful process of overcoming anxiety. This was observed in the descriptions of the 15% (3/20) of participants who already felt safe in their local environment and were unsure about the value of going further afield. These participants were in the *Struggling on* and *Hard to say* subgroups*.* For some, the relationship with the staff member was an important motivator to put into practice the learning from VR.

Through practicing in VR, participants described creating new learning and finding new explanations and understandings about themselves, other people, and the world. A common realization was that reality was not as bad as participants feared. The seed for these new beliefs was often found in VR—opportunities to make this discovery were embedded in the VR scenarios, for example, when characters smiled during interactions or simply did not respond as feared (eg, laughing or criticizing the participant). This new learning was tailored and reinforced by the staff member, ensuring that the key messages were personalized, succinct, and easy to recall. This learning often became most powerful when participants grew confident enough to push themselves in real life, making eye contact or small talk with people. These layers of corroboration, including discovery in VR, discussion with the deliverer, and real-world practice, made for a powerful learning experience. As well as embedding new beliefs, participants held onto memories created during gameChange. Some spoke of recalling scenes in VR or of positive experiences they had had afterward in the real world. This had a calming effect when they found themselves in a challenging situation:

It’s like [the staff member] says, “stand confident and people will see you as confident. No one is looking at you. People do look, it doesn’t say they’re going to hurt you.” It really helped me, because when I went out I thought of the gameChange.

Participant 21, Better ways to cope subgroup

The result of this practice and learning was that some participants saw changes that were important to them. The world had opened up for people whose lives were most restricted by agoraphobic avoidance:

I’ve been out on a couple of nights out and yeah, I’ve loved it really. Instead of hiding from it, I’m actually embracing it, I’m enjoying spending time with my friends because like I said I didn’t see them for a long time in the first lockdown because of my mental health and the state of mind I was in, it’s great seeing them now.

Participant 8, Big changes subgroup

It’s a case of not thinking twice about getting the bus and getting take away coffee now. I’m quite happy to do it which was a big change for me.

Participant 2, Big changes subgroup

## Discussion

### Principal Findings

Facilitated by peer methods, participants provided rich and informative accounts of the problem of agoraphobic anxiety and avoidance, the experience with automated VR therapy, and its impact on their lives. The restriction caused by severe agoraphobic avoidance has a substantial cost in people’s lives. Variations in the benefits of VR therapy were observed across the participant groups. Participants who were close to housebound (4/20, 20%) tended to report “big changes” from the gameChange VR therapy, whereas individuals who were reasonably comfortable with the boundaries of the locations that they could visit (5/20, 25%) tended not to report so much impact from the VR sessions*.* This perspective from the qualitative interviews of variation in benefits of VR therapy was gained before the trial outcome results were known by those involved in the qualitative analysis but were entirely consistent with the main trial findings. The largest group identified (9/20, 45%) derived benefit from gameChange—they developed “better ways to cope”—but in a less transformational way than the group who saw “big changes.” Their accounts suggest that, before the intervention, they were more able to perform everyday activities than the other group, but they were still distressed by their agoraphobia. These results support the conclusion that the implementation of gameChange is best focused first on people with the most severe agoraphobic difficulties.

There was further consistency with the quantitative findings. Participants reported a wide range of fears contributing to agoraphobic avoidance, as has been found using self-report assessments [4]. Furthermore, the participants viewed VR as a popular treatment option with limited mention of any side effects, which is consistent with our surveys across the whole group that received gameChange [23]. However, the interviews provided greater depth on several important issues. They provided an intriguing perspective on the therapeutic nature of immersive technology. Participants reported how VR—as they knew they were experiencing simulations—provided the chance to gain distance on anxious responses and try new alternative ways of thinking and behaving. Therapeutic gains arose out of a delicate balance of experiencing typical anxious responses but only to a degree in which safety could also be kept in mind. In this way, safety feelings could begin to feel like a realistic appraisal of the situations. This calibration was largely determined by the users, for example, selecting the scenario or level within the program, but there could also be invaluable support from the staff member who was present. A degree of guided support, even with automated therapies, is likely to be very important. It was also clear that participants considered that, to make change, it was important to apply the learning to real-world situations via the homework tasks. Conversations with the supporting staff member were valued. A common realization among those who gained benefits was that reality was not as bad as they feared. There are opportunities to further tailor the treatment to the individual by increasing the range of scenarios available and adapting the demographics of the virtual coach.

### Conclusions

The interviews provided fascinating in-depth insights into the experience with automated VR therapy, which will inform the delivery of gameChange alongside other implementation and health economic studies [23-25]. This study has several limitations. The participants interviewed were not representative of all those who received VR therapy. For instance, we only succeeded in interviewing participants who completed therapy, which is largely a reflection of the high uptake of VR therapy in the trial. This means that we would have missed important specific challenges and barriers for individuals who did not complete VR therapy. The participant group was also not representative of the wider population of people attending mental health services. There was a lack of ethnic diversity in the participants interviewed. Understanding the potential barriers faced by underserved and underrepresented groups will be important for future implementation. We also note that the interviews and therapy were conducted during the COVID-19 pandemic. The context of restrictions, disruption, and anxiety may have affected the experience of agoraphobic avoidance and VR therapy. Finally, the lengthy interviews that covered multiple questions mean that it is likely that we may only have been able to capture a fraction of participants’ experiences in this report.

## Appendix

Multimedia Appendix 1 Interview schedule for the gameChange experience study.

Multimedia Appendix 2 Illustrative quotes by superordinate and subordinate theme.

## Abbreviations

IPA: interpretative phenomenological analysis
LEAP: Lived Experience Advisory Panel
NHS: National Health Service
VR: virtual reality
