# Supplementary material for: A Safe Place to Learn: Peer Research Qualitative Investigation of gameChange Virtual Reality Therapy
Source: JMIR Serious Games. 2023 Jan 16;11:e38065. doi: 10.2196/38065 (PMC9947847; doi:10.2196/38065)
Supplement: Multimedia Appendix 1 [file games_v11i1e38065_app1.docx]

**Interview schedule for the gameChange Experience Study**

**Introduction**

**Just to start things off, can you tell us a bit about yourself?**

*Probes:* Perhaps what you like doing? What you have been up to this week?

**Had you come across the concept of virtual reality before getting involved with gameChange?**

**Had you experienced virtual reality before?**

-What did you think of it?

-[If they mention gaming] Is this something you do a lot? [probe further]

-Did you know it could be used for therapy? [If yes, probe what they thought about that before; had they used it therapeutically before?]

**What made you decide to try the VR gameChange therapy?**

*Probes*

- Was it suggested to you by one of your care team?

- Hopes / expectations

-Views of others (e.g. friends / family)

**Anxious social avoidance**

As you know, gameChange is about helping people overcome anxiety in everyday social situations or in public places.

**What is your experience of going out of the house or going into social situations/ public places?**

**Are there any situations that you find difficult?** **What makes them difficult?**

**What do you do when you end up in that situation?** (avoiding/not going, leaving early, taking precautions when there)

**What happens if you are faced with that kind of situation?**

*Probes:*

- What thoughts might you be experiencing? What runs through your head? Do you get any pictures of what might happen?

- How does it feel?

- Does it affect your body? /Do you notice any changes in your body?

**Is this something you have always experienced?**

*Probes:*

*-* If not, can you remember when you became aware of it?

**- Have you noticed any connection between social situations and your psychosis symptoms/worries about others/voices?** [Use participants own language and info from the local trial coordinator]

*Probe:* For example, do you feel more anxious about social situations when you are also experiencing other kinds of symptoms?

**How do these difficulties with social situations impact your life?**

*Probes:*

-Stopped you from going to certain places, seeing people, prevented you from doing things you wanted to

-Is it better or worse in some situations than others?

**Would life be different if you didn’t experience this? How?**

**What has the impact of coronavirus and the lockdown been on you in terms of anxieties about social situations?**

**How are you feeling about the restrictions easing?**

**Life before**

**If its ok with you, we’d now like to talk a bit about your life before you started gameChange.**

**Can you give us an overview of what was happening in the months before you met the gameChange team?**

*Probes*

-Lockdown

-Life in general (work, study, family, friends, hobbies, housing, finance)

-How long had you been experiencing symptoms for by then? [probe to get **at stage**: prodrome, newly diagnosed, recently relapsed, recently hospitalized, discharged from the CMHT, and **overview of treatments tried**)

-What was going on with your anxiety about social situations at that time? [if this isn’t clear above]

**Earlier you said you had xx hopes for the therapy,** d**id you have any concerns about the VR therapy beforehand?**

*Probes*

-Questions / doubts

**Life after the therapy/Impact**

**Before we talk about the therapy itself, we like to hear about what your life has been like since having the therapy.**

**Compared to what you just told us about your life before gameChange, is life different in any way now? How so?**

*Probes:*

-Have you noticed any changes since doing gameChange?

-How did you feel about social situations after treatment compared to before?

-Have you been able to do things that you weren’t able to do before? What things?

-Have you gained more understanding of where your fears come from or how they affect you? Can you tell me more…

-Have there been any other improvements or any negative effects? Such as…

-[if needed] For example, do you think you are less anxious in everyday social situations now?

-Anything that you thought might change but hasn’t?

**Why do you think this might be?** (probe to get at mechanism for whatever the change or lack of mentioned)

What do you think helped this change to happen? Did anything get in the way of change occurring?

**Looking back, was there anything going on in your life at the time that might have affected your experience of the therapy?**

*Probe:* life events, family / relationships, other activities or changes taking place at the same time, health

**The therapy**

**This part of the conversation is all about your experience of the gameChange therapy itself.**

**What was it like to do the VR therapy?**

*Probe:* How did it feel to be in virtual reality? What did it make you think?

**Can you walk us through a typical session?**

**What was your experience of the different VR environments?**

*Probe:* Did anything particularly stand out for you? Why?

**What was your experience of the virtual people that you interacted with?**

**Were any of the environments relevant to your daily life? Or to the things (places / situations) that you tend to avoid?**

**What was this like to be in this situation in virtual reality?**

*Probes:*

-Did it feel real?

-What emotions were you experiencing? What were you thinking about?

-Did it feel frightening?

-What did you take away from the experience? For example, did you discover anything new in the VR world?

-Were you able to do anything in the VR world that at the time you weren’t able to do in the real world?

-When you were in the VR world how did you cope?

Did you find yourself using the same coping mechanisms, [remind what these were] as you described earlier?

**Did you feel you had a say in how the therapy went?** /Did you feel you had some control over how the therapy went?

-For example, the scenarios you explored, the pace you went at, any goals that you set for yourself?

- How did you choose the scenarios, and when to move through the different levels? Did you talk about this with the delivery staff?

**What was your experience of Nic the coach?**

*Probe:* What was it like to have a virtual coach to talk you through things?

What worked well? What didn’t?

**What was your experience of the person who was in the room with you?**

*Probes:* What sort of things did they help you with? [reinforce, set homework]

Did they help you with the kit?

Were there any issues related to the kit or the VR technology? (probe to get at problem and solution, how this shaped their relationship)

What worked well with them there? And what didn’t?

How did you decide what pace to go at/when to move up a level?

When discussing this, did you and the deliverer always agree?

Were there times when they were encouraging you to try something and you were unsure/reluctant/didn’t want to? (probe to get at dynamic of SU resistance/avoidance and need for exposure/challenge)

Did they help to make the therapy ‘fit’ you better, either in terms of adjusting the equipment (or times of day, routines whilst doing it, etc), or going through in a specific order?

Was anything else done to adapt the therapy to you?

**What was it like to have someone outside the virtual environments and Nic inside them?**

**How did you feel straight after the VR session?**

*Probes:*

-What did you tend to do straight after a session?

-Would you talk to anyone about it?

**What was it like in between sessions?**

-Did you think about your last session or anticipate the next one?

-Did you do anything in between as a result of the previous session?

-Did you have homework? Was this helpful? What worked with the homework? Was there anything that didn’t?

**Have been able to take what you learned in VR to the real world? [If no] What made this difficult?**

**Were you having any other treatment at the time or seeing any other healthcare professionals? How did gameChange fit in with this?**

**Did you have any concerns about doing gameChange during coronavirus?**

**Do you think the experience would have been very different if there wasn’t a pandemic?**

**Overall experience**

**Thinking about your overall experience of gameChange now, what did you like about it?**

**What did you not like about it?**

**Would you change anything about it?**

*Probes:*

-This can be in any way, including the VR therapy itself so the types of situations, homework, or your experience of the VR equipment, but also everything else about it - the location of the sessions, the process of getting to them, the number of sessions etc. [Probe for this if they don’t bring it up]

**How does gameChange compare to other treatments you have tried?**

-Have you thought about trying other treatments for psychosis / social anxiety that you have heard of? [If yes] What attracts you to them?

*-*Would you recommend gameChange to others?

**Have you told others (e.g. your friends, family or carer) about gameChange? What did you say to them?**

*Probes:*

-What did they think about it?

-Do you think their opinions have influenced your perspective on it?

[if didn’t talk about it with others] **Why didn’t you? How would you describe taking part in gameChange to a friend?**

**Given everything we have talked about, is there anything else that you think is important for us to know about?**

**END**
