# Supplementary material for: A Safe Place to Learn: Peer Research Qualitative Investigation of gameChange Virtual Reality Therapy
Source: JMIR Serious Games. 2023 Jan 16;11:e38065. doi: 10.2196/38065 (PMC9947847; doi:10.2196/38065)
Supplement: Multimedia Appendix 2 [file games_v11i1e38065_app2.doc]

**Table S1.** Illustrative quotes by superordinate and subordinate theme.

| Theme and subtheme^a^ and  Participant subgroup | | | Participant identifier | Quote |
| --- | --- | --- | --- | --- |
| **Experience and cost of anxious avoidance** | | | | |
|  | Big changes | | 8 | “Even opening the curtains, letting light in, little things would give me anxiety so I would just be in my room, curtains closed just on my laptop just being like a waste of space really.” |
|  | Better ways to cope | | 12 | “I should be able to go for a walk. I should be able to just get on a bus. I should be able to go shopping without planning it with days in advance. I should go and sit down and have a coffee and do whatever, but I just can’t because this anxiety it goes through the roof. So yes, my life would be very different, definitely and I’d probably see my friends more as well.” |
|  | Hard to say or hard to hold onto | | 17 | “I just get really tight-chested and sometimes I just feel like, when it happens, I get really frustrated with myself because I have to try to talk to myself and say, nothing is going to happen, it’s alright but then quite often when I get really anxious, I just feel like I can’t go on like this, this is stopping me living my life. This is no kind of life for me, and I can have quite negative thoughts.” |
|  | Struggling on | | 19 | “I can just about handle being in the locality and going to my local pub and going for local walks, but that’s all that I do. …..So, yeah, I have a very limited life really, but it’s safe and it’s secure.” |
| **Reasons to try: *curiosity and motivation*** | | | | |
|  | Big changes | | 1 | “At the end of last year, I was determined this year that I was going to make a change in my life and then go out more and address my anxieties and things. And so, it fitted in really, really well with that.” |
|  | Better ways to cope | | 12 | “At one time I was like, “‘No, I don’t want to do this, I don’t want to do that,’” and now I want to learn... I’ve got a little girl to look after and I need to find techniques and learn more about what’s going on.” |
|  | Hard to say or hard to hold onto | | 5 | “The last couple of years I’ve been trying to get just to see a psychologist and stuff so I was pretty up for trying anything really because I’ve had such a long wait with my default sort of care and I thought it would be interesting.” |
|  | Hard to say or hard to hold onto | | 7 | “I knew that it was definitely the start of something new because I know the gaming industry is geared towards virtual reality and that and trying to refine it and make it as realistic as possible and stuff. It probably is the future for gaming.” |
|  | Hard to say or hard to hold onto | | 13 | “To be honest, the main reason that I tried gameChange, I’ll be honest, it wasn’t for therapy, it was because I was interested in seeing how it [the VR] was, to be honest, it wasn’t because that I thought it was going to drastically transform myself. I did it because I mean, I found it fascinating.” |
| **A place to practice: *an immersive experience*** | | | | |
|  | Big changes | | 27 | “It was really cool, it felt like you were in the game, but you wasn’t, you were in real life, and you was trying out new things in this world and then you could take them and put them into real life situations.” |
|  | Better ways to cope | | 21 | “It’s actually real, you feel, not in the world as we are now, the world we’re in without the headgear on. But when you go into the headgear, that is the world. It’s actually real. I mean, you see people passing, talking. And they are… ...they don’t seem as though they’re not real, you know.” |
|  | Better ways to cope | | 3 | “It seemed like real life. You know, everything, all the graphics, everything was really high, high spec. So, it was easy to relate to a real-life situation when I was in the VR.” |
|  | Struggling on | | 26 | “It’s like you’ve got this computer world. You’ve got, like, a different world. It seems like real life but it was a bit different from real life. It mimics real life.” |
| **A place to practice: *a chance to observe anxiety*** | | | | |
|  | Big changes | | 1 | “I didn'’t expect to start getting palpitations and things like that. But I actually did a few times. But that was good because I managed to then talk about and practice, like, sticking with it and staying in that situation rather than running.” |
|  | Better ways to cope | | 12 | “When I went into the VR, I didn’t realise that I would have such bad anxiety because I’ve got to do certain things anyway.  Like go shopping, go to school in the queue, wait for her…  ...Yes, it was more difficult than I expected.” |
|  | Better ways to cope | | 23 | “When I first did it I was a bit shocked, obviously because I didn’t realise that I had been doing things like that. Not taking notice.” |
|  | Hard to say or hard to hold onto | | 5 | “Now that you notice those [anxiety] things, are you able to do anything about them?”  “I guess it’s just trying to understand it’s not something that’s going to last forever anyway so it’s up and down. It’s trying to ride through it.” |
|  | Hard to say or hard to hold onto | | 17 | “It really made me realise how anxious I get being outside and it made me feel a bit upset as to why do I feel like that when other people can just walk down the street.” |
| **A place to practice: *new ways of responding*** | | | | |
|  | Better ways to cope | | 16 | “The person that was supporting me in the VR, she told me that I can show my symptoms and nothing bad will happen to me, so I showed my symptoms, this was a task that she set up especially for me in the VR, and afterwards, I found out about myself that I am more confident than I think.” |
|  | Better ways to cope | | 3 | “So, the supermarket would be a case of going in, so slowing everything down looking on the shelves rather than just getting what I wanted and then going; it was a case of looking at other things as well that I didn’t need or didn’t want, and then looking at people while I was in the shop rather than just being in and out.” |
|  | Better ways to cope | | 15 | “She was like: “‘You need to stand like you’re a confident person, stand like you’re confident and you might feel a bit more confident,’” so I really tried to do that throughout it as well.” |
|  | Hard to say or hard to hold onto | | 11 | “The [strategy] one that she taught us was try and look at something and describe things about it. So if I’m in a café or something and I’m paranoid, then look into a sandwich or something and describe what’s in the sandwich.” |
| **The security of knowing it’s not real: *the sweet spot of safety and anxiety*** | | | | |
|  | Big changes | | 8 | “It felt pretty real. However, like in the back of my mind, I knew that it was obviously just VR and to me to distinguish that and the real world, for me personally, I guess I knew I could always escape in a way.” |
|  | Better ways to cope | | 15 | “I was expecting [the VR] to be a little bit more real than it was, which I think is a good thing because then you can differentiate between… ...you can definitely know that it’s not real.” |
|  | Hard to say or hard to hold onto | | 5 | “I guess it could have been better [the VR] but then again, if it’s meant to be the stepping stone or the training wheel, you want it to be not so realistic or anything.” |
|  | Hard to say or hard to hold onto | | 13 | “I knew that the people in the VR didn’t have their own thoughts and opinions, but when you’re in the VR some of them can stare at you and stare at you for an unusually long amount of time. That can feel very like…...it can make you feel uncomfortable sometimes, I must admit.” |
| **The security of knowing it’s not real: *calibrating for a personalized approach*** | | | | |
|  | | Big changes | 1 | “I liked Nic [the virtual coach] to start with and then I found her very, very annoying. But just because she was saying the same speech at sort of the beginning of each thing.” |
|  | | Better ways to cope | 26 | “She [the virtual coach] was talking me through things, explain what’s going to happen and talk me through things. Being encouraging to you...…It’s like having a friend there really.” |
|  | | Better ways to cope | 21 | “[The deliverer] would talk me through it. She’d say like, “‘Oh, a man’s going to… ...look behind. Turn around, you’ll see a man.’” I’ve looked, oh, yeah. I’ve seen a man. And things like that, it guided me through it. If I had been on my own, I think I would have problems. I’d be more nervous and jumpy with it.” |
|  | | Better ways to cope | 15 | “She’d notice if I looked like I was more anxious about things, that was quite helpful to acknowledge that. Maybe she’d say: “‘Why don’t you look round and just look at all the art on the wall in the room, or why don’t you count the tiles on the floor?’” or something like that and that was quite helpful.” |
|  | | Hard to say or hard to hold onto | 17 | “She could tell if I needed a break as well. So, she would… ...you know, I could say any time. Sometimes I felt that I needed to carry on. But she was really good and she'’d say, you know, “‘Shall we take a break and then talk about it and go back?’” |
| **Taking it into the real world: *from training wheels to real-world practice*** | | | | |
|  | | Big changes | 8 | “That probably helped me the most really, just making notes on each session. Those notes I would make would be because of me asking the researcher therapist questions, she’d tell me things to write down, and I’d just write it down and then yeah, go from there.” |
|  | | Big changes | 2 | “I tried to be as proactive as possible really. I certainly didn’t think it was a case of go along for the session and that’s it, don’t do anything else until the next one. Even if, as I said with the café one and the pub one, you couldn’t really practice [due to Covid], you could still think about what you’d been through in the virtual world and how it might make you change your way of thinking in the real world when the situation arises.” |
|  | | Big changes | 1 | “Every week, I had at least one thing to do before the next session. And I did find it really useful to have it written down and not be held accountable, but to, you know, not to have infinite amount of time in which to do whatever it was. So, that was good that I had to do it sort of within a week.” |
|  | | Better ways to cope | 25 | “[In between sessions] I was thinking about completing that goal, so thinking about the last session I’d done, and what I’d learnt in the last session that I could carry through to my goal.” 25 |
|  | | Hard to say or hard to hold onto | 7 | “I didn’t really want to let her down and I didn’t want to lie to her, so I did it.” |
| **Taking it into the real world: *one thing to hold onto*** | | | | |
|  | | Big changes | 1 | “I stood outside and had a smoke on the other side of my gate, which I was having a problem getting beyond, which felt really, really strange and weird. But the second time I did it, I met a neighbour and chatted, chatted to a few neighbours. And they were quite friendly. And it was a nice experience, which isn'’t at all what I was expecting or even worrying about.” |
|  | | Big changes | 8 | “If I think someone looked at me funny or if I think someone’s looking at me and laughing, acknowledge the thought like “‘okay, that person is looking at me and just let it go because it doesn’t matter.’” ... Everyone looks at everyone and that’s all right [laughter].” |
|  | | Better ways to cope | 12 | “I even talk to the guy at the till because I’ve practised that a few times and it was hard at first but then I was like, well why was I so bothered about that?” |
|  | | Better ways to cope | 3 | “I used to say at the end of a session, oh, “‘It wasn’t as bad as it seemed,’” or, “‘It wasn’t as bad as I thought,’” so then she would use my words and like say, “‘How would you feel about trying this? Because that mightn’t be as bad as you think.’” So, I think that’s a good example in terms of what [the deliverer] did to bring me on.” |
|  | | Hard to say or hard to hold onto | 17 | “So, saying that anxiety is not a predictor of what’s going to happen, about being physical, so even if you’re feeling anxious and you’re not feeling confident, do the opposite. So, if you’re feeling like, I want to look at the ground, I don’t want anyone to look at me, you just stand up straight and walk purposefully and just act confident, even though you’re not feeling confident.” |
|  | | Big changes | 2 | “What I did find was walking into town, for example, that would be very useful to think about what that virtual world was like and with the people passing you and all that sort of thing, it helps to calm down the anxiety feelings that you might have.” |
|  | | Better ways to cope | 21 | “If I go to the shop now, say there was six people in, I’d think of what the video was like at gameChange, people talking amongst themselves, getting food, drink, I just think that’s all the other shoppers are doing and it has helped me.” |
